# Host-associated and Environmental Microbiomes in an Open-Sea Mediterranean Gilthead Sea Bream Fish Farm

**DOI:** 10.1007/s00248-022-02120-7

**Published:** 2022-10-07

**Authors:** Grazia Marina Quero, Roberta Piredda, Marco Basili, Giulia Maricchiolo, Simone Mirto, Elena Manini, Anne Mette Seyfarth, Marco Candela, Gian Marco Luna

**Affiliations:** 1 Institute for Marine Biological Resources and Biotechnologies, National Research Council (CNR-IRBIM), Ancona, Italy; 2 Department of Veterinary Medicine, University of Bari Aldo Moro, Valenzano (Bari), Italy; 3 Alma Mater Studiorum—University of Bologna, Bologna, Italy; 4 Institute of Marine Biological Resources and Biotechnologies, National Research Council (CNR-IRBIM), Messina, Italy; 5 Institute of Anthropic Impacts and Sustainability in Marine Environment, National Research Council (IAS-CNR), Palermo, Italy; 6 Department of Global Surveillance, National Food Institute, Technical University of Denmark, Lyngby, Denmark

**Keywords:** Gilthead seabream, Fish microbiome, Aquaculture sustainability, Mediterranean Sea

## Abstract

Gilthead seabream is among the most important farmed fish species in the Mediterranean Sea. Several approaches are currently applied to assure a lower impact of diseases and higher productivity, including the exploration of the fish microbiome and its manipulation as a sustainable alternative to improve aquaculture practices. Here, using 16S rRNA gene high-throughput sequencing, we explored the microbiome of farmed seabream to assess similarities and differences among microbial assemblages associated to different tissues and compare them with those in the surrounding environment. Seabream had distinct associated microbiomes according to the tissue and compared to the marine environment. The gut hosted the most diverse microbiome; different sets of dominant ASVs characterized the environmental and fish samples. The similarity between fish and environmental microbiomes was higher in seawater than sediment (up to 7.8 times), and the highest similarity (3.9%) was observed between gill and seawater, suggesting that gills are more closely interacting with the environment. We finally analyzed the potential connections occurring among microbiomes. These connections were relatively low among the host’s tissues and, in particular, between the gut and the others fish-related microbiomes; other tissues, including skin and gills, were found to be the most connected microbiomes. Our results suggest that, in mariculture, seabream microbiomes reflect only partially those in their surrounding environment and that the host is the primary driver shaping the seabream microbiome. These data provide a step forward to understand the role of the microbiome in farmed fish and farming environments, useful to enhance disease control, fish health, and environmental sustainability.

## Introduction

Aquaculture is the fastest-growing–food-production sector in the world and is predicted to contribute to more than half of the fish consumed globally by 2030 [1]. Gilthead seabream (*Sparus [S.] aurata*) is among the most important fish species farmed in the Mediterranean Sea [2, 3], with production increased by 77% over the last decade [4, 5] and with the EU representing the biggest producer worldwide for this species (https://ec.europa.eu/fisheries/sites/fisheries/files/docs/body/sea-bream_en.pdf). Aquaculture, including seabream farming, poses serious environmental concerns [6], represented mainly by organic wastes release into the surrounding environment, compromising the water quality and the benthic ecosystem nearby [7]. In addition, fish farms often experience diseases outbreaks due to microbial agents [8, 9], which cause further economic losses. Several solutions have been tested in order to assure a lower impact of farmed fish diseases while guaranteeing a higher productivity, most of them being based on the use of antimicrobials or vaccines [10, 11]. Solutions are likewise being used to reduce the organic enrichment of farm sediments by bioremediation actions [2]. An overall safer, more sustainable production of farmed fish is today demanded at the global level and developing under the principles of One Health to achieve beneficial health and well-being outcomes for people, non-human organisms, and their shared environment [12].

In this perspective, research efforts are being increasingly performed to improve the quality of farmed fish production by the exploitation and modulation of the fish microbiome [8, 9, 11, 13]. To do this, an improved and complete understanding of the teleost microbiome is required. However, while in the last years several works have been published on the fish microbiome, thanks to the application of high-throughput sequencing (HTS) [13], our knowledge is still in its infancy [13]. Most of the recent studies have been devoted to study fish gut and/or feces microbiome [14–16] with less works exploring at the same time the skin, gill, and other tissue’s microbiomes [3, 17, 18]. Also, it still remains unclear how microbiome diversity is partitioned among the different niches within teleosts, and how this partitioning might potentially be linked to the fish health.

Learning more about the microbiome of fish is essential given that, as for humans and other animals, the fish microbiome is crucial in protecting the health status of its host and in boosting the performance of the host immune system, well-being, food digestion, and synthesis of vital nutrients [8, 13, 18]. Mucosal surfaces such as skin and gills act as primary barriers to disease [3, 17–19] and play a key role in fish innate immunity [20]. At the same time, the gut microbiome has an active role in modulating the host’s physiology, assisting the intestinal development and physiology, as well as the overall development, growth, health, energy homeostasis, and immune response of the fish host [21]. Along with host-related benefits, the fish microbiome is increasingly being recognized as a means to obtain safe and high-quality food products for human consumption; understanding the prevalence and ecology of microorganisms, including those pathogenic and spoilage, associated to the fish tissues (e.g., skin, gill, gut) and products (e.g., fillet) along the production chain, has been also recently suggested to contribute to the development and application of new intervention strategies [9, 22] and a step forward to address the One Health concept also in aquaculture [12].

Among the drivers of the fish microbiome, diet is known to exert a major impact with particular reference to the gut [8], but also host-related factors, such as fish hosts’ genetic features, can play a role. Inter- and intra- host genetic diversity can drive the inclusion of beneficial or neutral microbes and the exclusion of potential pathogens [23]. However, the composition of the fish microbiome can also be affected by the environmental microbial communities, such as those associated with seawater and sediment [8]. Several studies have been performed on the inner (gut) and outer (gill and/or skin) fish microbiomes, with findings generally supporting the view that the environment is a source from which fishes acquire their microbiome [8, 16, 24, 25]. This aspect has not yet been clarified in Mediterranean sea bream, where information on the interactions between this species and the surrounding environment has been assessed only in early stages reared in tanks [26]. Moreover, it still remains under debate whether, and to what extent, the marine environment represents a source or a sink of the teleost microbiome in different compartments. To the best of our knowledge, no studies have been conducted in open mariculture settings to simultaneously assess the environmental microbiomes (sediment and seawater) and those associated with different seabream body tissues such as gut, skin, and gill; in addition, data on the presence, diversity, and abundance of microorganism in fish muscles (fillet) is still underexplored and up to debate.

In this study, by means of HTS 16S rRNA data, we aimed at exploring the microbiome of farmed Mediterranean gilthead seabream, the main farmed fish in the Mediterranean Sea, by (a) analyzing and identifying differences in the skin-, gill-, gut-, and fillet-associated microbial assemblages; (b) comparing the fish microbiome to environmental microbial communities (i.e., seawater and sediments); and (c) deciphering the potential connections occurring among microbiomes. Our study provides a step forward to the understanding of the diversity of the farmed sea bream microbiome in relation to the different fish tissues and the surrounding environment and aims at supporting future actions for enhancing disease control, fish health, and environmental sustainability in open-sea aquaculture settings.

## Material and Methods

### Study Area and Sampling Activities

Sampling activities were performed on 25th September 2019 at a marine fish farm located in the harbour of Licata (Sicily, Mediterranean Sea; coordinates 37.087713° N, 13.943773° E), within a semi-enclosed and sheltered area, characterized by a limited hydrodynamic circulation and a shallow depth (~ 10 m); further details on the sampling site are reported in [2] and [27].

Five adult individuals of *S. aurata* (avg. 273.2 g ± 32.3 g) fed a commercial diet were collected by a fishing net from a single sea cage. Fish specimens were immediately euthanized with a lethal dose (0.5 g/L) of Tricaine methanesulfonate (MS222), according to the ethical principles and national legislative context, and placed on ice until arrival at the laboratory around 1 h later; weight, total, and standard length were measured. Fish specimens were dissected within 4 h of capture.

For each fish specimen, we isolated: (1) fish skin (a 2 × 2 cm square from the left size), (2) fish gill (second gill arch on the left gill), (3) fish gut (fore-, mid-, and hindgut were separated; feces or digesta were collected by gentle squeezing, mucosae were collected by scraping of the internal side of the gut tube, and remaining gut tissue was also collected), (4) fish fillet (2 × 2 cm square at full depth from the left side). Except for fish feces, all the other tissue samples were rinsed with sterile phosphate buffer solution to remove possible loosely attached microorganisms. All described types of samples were obtained by dissecting fish specimens using sterile scalpels and scissors, immediately placed in sterile tubes, and stored at − 20 °C until further analyses.

Surface seawater and surface (0–1 cm) sediment samples were also collected at three sites, including the seabream sampling cage (37.086667°N, 13.943611°E) and at two additional sites (37.089732°N, 13.937469°E, and 37.091949°N, 13.933703°E), using a sterile plastic bottle and plexiglas cores, respectively. Further details on seawater and sediment sampling are reported in [27].

### DNA Extraction and Sequencing

Seawater samples (one liter) were filtered onto 0.22 μm cellulose nitrate membrane filters (Sartorius, ø 47 mm) and stored at − 20 °C until processing; the entire membrane filters were used for DNA extraction. One g of the top 1 cm of sediment cores previously extruded and stored at − 20 °C was used for DNA extraction. DNA from sea bream skin, gill, fillet, feces and gut tissues, seawater, and sediment samples was extracted using the DNeasy PowerSoil Kit (Qiagen) as previously described in [27]. Extracted DNA samples were quantified with NanoDrop ND-1000 (NanoDrop Technologies, Wilmington, DE, USA) and stored at − 20 °C until processing. The PCR amplification of the V3-V4 hypervariable region of the 16S rRNA gene was carried out using the primer pair 341F-785R [28] and the PCR product purified as described in [27]. Nextera library indexing and preparation and Illumina MiSeq sequencing (2 × 300 bp paired-end protocol) were performed as described in [27]. The obtained raw sequences are submitted to the SRA—Sequence Read Archive (BioProject PRJNA692072).

### Data Analysis

Primer and adapter sequences were removed from raw reads with Cutadapt [29]; paired-end reads were then imported and analyzed in RStudio (RStudio Team, 2020) using the DADA2 package [30]. Quality check and trimming of the reads were performed following the package instructions (at 280 and 230 bp for forward and reverse reads, respectively; max estimated error higher than 2 and 2 per 100 bp for forward and reverse reads, respectively). Paired-end reads were subsequently merged in amplicon sequence variants (ASVs, i.e., clusters sharing 100% sequence identity); chimeric sequences were identified and removed from the dataset. Finally, prokaryotic taxonomy was assigned using a native implementation of the naive Bayesian classifier method against the *silva* database (v132; https://www.arb-silva.de/documentation/release-132/). For alpha and beta diversity, all libraries obtained from gut samples (i.e., fore-, mid-, hindgut tissues, mucosae, and feces) were pooled per each fish sample. Chloroplast and eukaryotic sequences were removed from the ASV table obtained from DADA2; the table was normalized to the lowest number of reads present among the samples (*n* = 13,121) with the *vegan* package [31].

All statistical analyses were performed in Rstudio (RStudio Team, 2020). For the analysis of alpha diversity, ASV richness was calculated using the *vegan* package [31]. The occurrence of statistical differences among richness values in the different types of samples was assessed with a Kruskal–Wallis test (*stats* package) considering all possible comparisons. Non-metric multidimensional scaling (nMDS) was performed using a Bray–Curtis dissimilarity matrix and average linkage approach and plotted with the *ggplot2* package. Significant differences in prokaryotic community composition between sample types (i.e., fish vs. environmental) as well as among matrices (i.e., skin, gill, gut, fillet, sediment, and seawater) and among fish tissues (i.e., skin, gill, gut, fillet) were calculated by using PERMANOVA through the *adonis* function (*vegan* package) in R, based on a Euclidean distance matrix calculated on the Hellinger transformed ASV table. A bubble plot including the most abundant ASVs within each sample was plotted using the *ggplot2* package [32].

With the aim of exploring the connections occurring between microbiomes associated to the different fish tissues and the surrounding environment and in order to preliminarily hypothesize the ecological meaning of such connections, we performed network analysis using the network-based analysis performed by the *make_otu_network.py* script in QIIME v 1.9.0 [33]. This approach is used to analyze and display how ASVs were exclusive or shared among samples, with the aim to emphasize taxonomic similarities and differences among microbiomes. The network data were imported in Gephi (www.gephi.org) for visualization; in the obtained plot, the more similar are the samples, the closer they are positioned in the plot. The Venn diagrams were calculated using the R package *Venn* (https://github.com/dusadrian/venn), and the results from this analysis were reported in a table, as described in the “Results” section.

## Results

### Prokaryotic Diversity and Community Composition

**Fig. 1:**
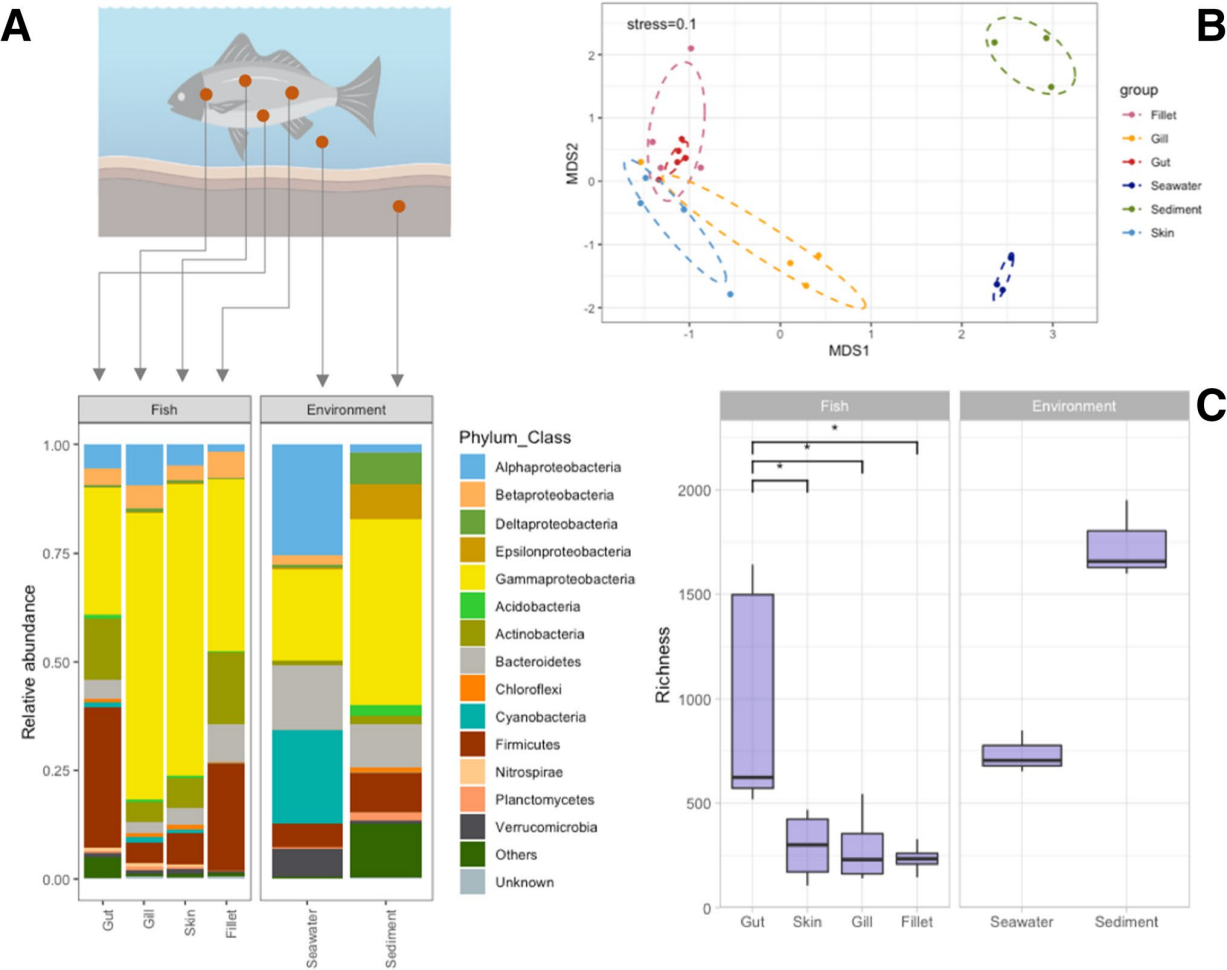
Panel **A**: barplot showing the prokaryotic community composition (as relative abundance) at the phylum and class level (for Proteobacteria only). Taxa with an average relative abundance across all samples < 1% were aggregated as “Others”. “Unknown” includes all those reads that did not match any known taxa. Panel **B**: nonmetric multidimensional scaling (NMDS) ordination of community composition of fish and environmental microbiomes based on Bray–Curtis dissimilarity matrix. Panel **C**: richness calculated for the different types of samples; asterisks indicate the occurrence of significant differences as calculated by the Kruskal–Wallis test (*p* < 0.05); non-significant comparisons are not reported in the plot. Richness in environmental samples also significantly differed from those observed in fish-associated microbiomes (Kruskal–Wallis *p* < 0.05). The fish figure has been created in BioRender (https://biorender.com/)

Taxonomic composition at the phylum level (Fig. 1A) highlighted the dominance of Proteobacteria in all samples; in particular, fish communities were particularly enriched in Gammaproteobacteria (avg. 49.24 ± 31.65%), followed by Alpha- (avg. 5.39 ± 6.67%) and Betaproteobacteria (avg. 4.61 ± 5.8%). Higher proportion of Alpha (25.03 ± 0.62% and 1.83 ± 0.27% in seawater and sediment, respectively), Delta- (0.71 ± 0.3% and 7.34 ± 1.53%), and Epsilonproteobacteria (0.22 ± 0.07% and 8.04 ± 10.48%) were overall observed in environmental samples. Fish samples also displayed high relative abundances of Actinobacteria (avg. 10.73 ± 10.56%) and Firmicutes (avg. 18.17 ± 14.19%), with particular regard to gut (14.27 ± 6.07% and 32.46 ± 11.07% for Actinobacteria and Firmicutes, respectively) and fillets (16.52 ± 18.44% and 24.73 ± 7.84% for Actinobacteria and Firmicutes, respectively) microbiomes (Fig. 1A). Cyanobacteria were observed mainly in seawater (avg. 21.63 ± 0.54%); higher proportions of Bacteroidetes finally characterized environmental samples (14.94 ± 0.92% and 9.76 ± 6.42% in seawater and sediment, respectively).

Non-metric multidimensional scaling (NMDS) (Fig. 1B) underlined the clear separation between fish and environmental samples, while showing a partial overlapping of communities belonging to the different fish tissue types. PERMANOVA analysis confirmed what previously observed for richness: microbiomes associated with fish tissues were significantly different from those in the surrounding environment (*adonis*, *p* < 0.001), while microbiomes associated with the different fish tissues, significantly differed among each other (*adonis*, *p* = 0.006).

Alpha diversity (i.e., ASV richness) (Fig. 1C) indicated an overall higher diversity in environmental microbiomes (i.e., seawater and sediments) than in those associated with fish, with sediment hosting the most diverse assemblages of the entire dataset. In more detail, statistical differences occurred between the number of ASVs harbored by fish- and environment-related samples (*p* = 0.004). Considering microbiomes from the fish specimens only, we observed a significantly higher diversity occurring in gut samples as compared to skin, gill, and fillet samples (*p* < 0.05) (Fig. 1C).

**Fig. 2:**
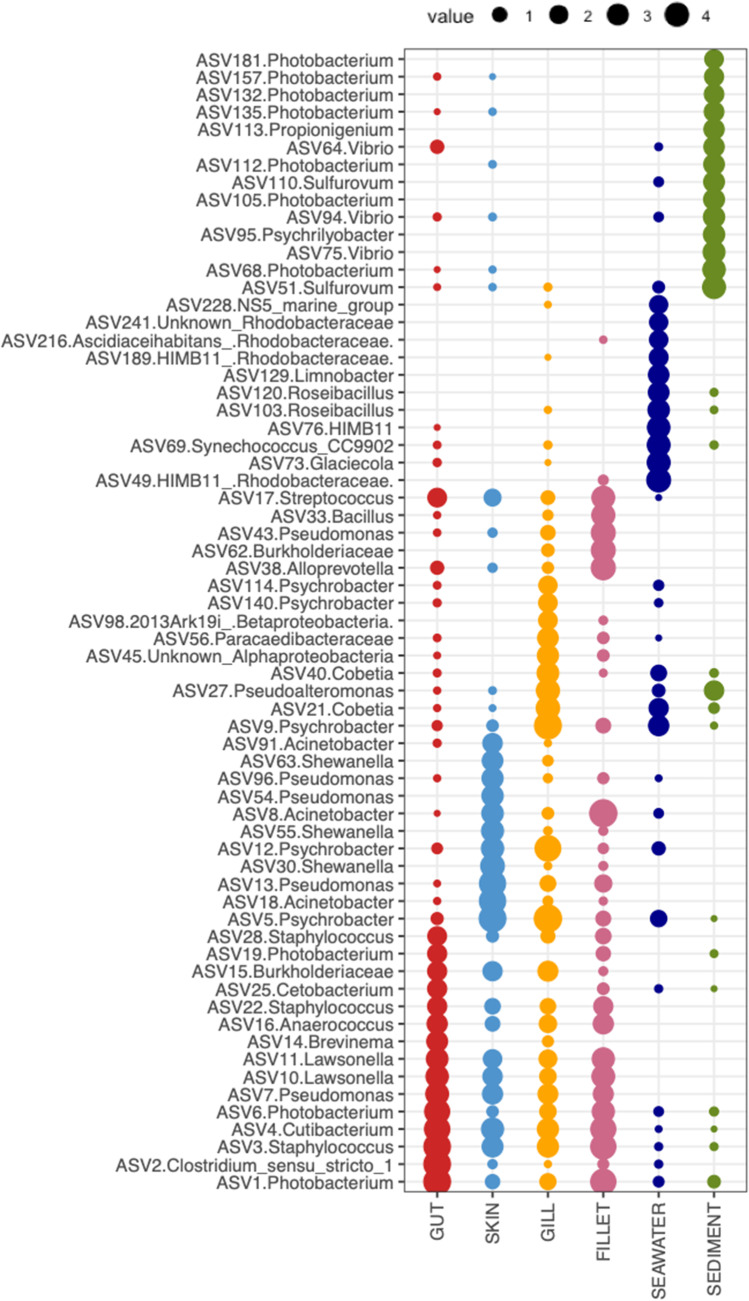
Bubble chart reporting the proportion and taxonomic identification of the most abundant ASVs (avg. > 0.5% across the dataset) observed in each fish and environmental microbiome. The dimension of each bubble is proportional to the log of the relative abundance of the ASV

The analysis of the most abundant ASVs represented in the different types of samples (Fig. 2) highlighted that different sets of abundant ASVs characterized the environmental and fish-associated microbiomes. ASVs identified at the genus level as *Photobacterium*, *Vibrio*, and *Sulfurovum* were particularly abundant in sediment samples, whereas the seawater microbiome was enriched in ASVs identified as Rhodobacteraceae, *Synechococcus*, NS5 marine group, *Limnobacter*, *Glaciecola*, and *Roseibacillus*. Fish gut samples contained, at high abundances, ASVs belonging to the genus *Photobacterium* but different from those observed in sediment and seawater; ASVs identified as *Streptococcus*, *Staphylococcus*, *Anaerococcus*, *Lawsonella*, *Pseudomonas*, *Cutibacterium*, *Clostridium*, and *Brevinema* were also abundant in these type of samples. ASVs assigned to the genera *Pseudomonas*, *Acinetobacter*, *Psychrobacter*, and *Shewanella* were present as the most abundant in fish skin samples; moreover, some ASVs identified as *Lawsonella*, *Staphylococcus*, *Pseudomonas*, and *Cutibacterium* appeared to be shared also with gut, gill, and fillet microbiomes. Gill samples were enriched in ASVs belonging to the genera *Psychrobacter*, *Cobetia*, and *Pseudoalteromonas*; even in this case, several ASVs were shared with the other fish microbiomes’ most abundant ASVs. Finally, fillet microbiomes displayed a set of abundant ASVs and mainly belonging to the genera *Streptococcus*, *Bacillus*, *Pseudomonas*, *Shewanella*, *Photobacterium*, *Staphylococcus*, *Lawsonella*, and *Cutibacterium*.

**Fig. 3:**
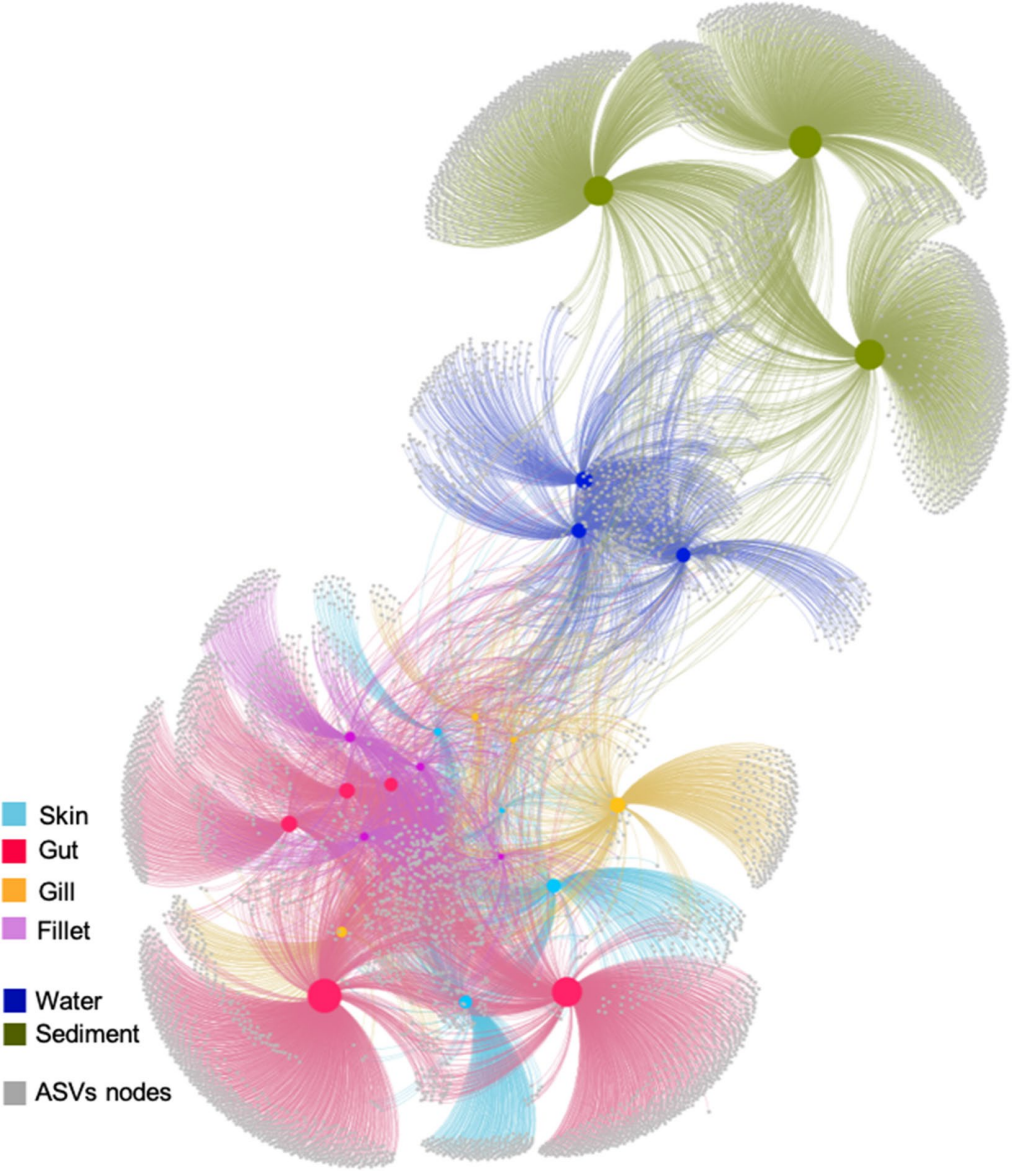
Network-based analysis to display and analyze how ASVs are partitioned between samples. In the network diagram, there are two kinds of “nodes” represented, ASV-nodes (grey) and sample-nodes (colored according to the type of sample: red, gut; yellow, gill; cyan, skin; pink, fillet; dark blue, seawater, green, sediment). The network has been calculated excluding singletons. Sample nodes’ size is proportional to their degree, i.e., the number of connections with ASVs nodes. Edges (i.e., lines between nodes) connect ASVs to the samples where they are present; shorter distances between the sample and ASV nodes reflect larger numbers of sequences from that ASV found in the connected sample. Finally, ASVs occurring in the center of the network are more evenly distributed between all the samples, while those on the edges occur preferentially in certain samples

We used the make_otu_network.py script implemented in QIIME in order to visualize how ASVs were shared among different fish tissues and environmental samples (Fig. 3). The results of this analysis showed that unique ASVs occurred in all fish tissues and environmental samples, with the gut being the tissue hosting the largest number of unique ASVs for fish samples (*n* = 1945, 73.8% of total ASVs for gut samples) and the fillet being the one with the smallest (*n* = 181, 30.8% of total ASVs for fillet samples). Notably, sediment samples included the highest number of exclusive ASVs for the whole dataset (*n* = 2486, 93.8% of total ASVs for sediment samples). More in detail, only six ASVs were found to be present in all samples (ASV5 *Psychrobacter*; ASV3 *Staphylococcus*; ASV1 *Photobacterium*; ASV4 *Cutibacterium*; ASV9 *Psychrobacter*; ASV6 *Photobacterium*) and, in general, the fish microbiomes shared a lower percentage of ASVs with the environmental communities from sediment (0.6–0.8%) than seawater (Table 1). Interestingly, the highest number of shared ASVs between fish and environmental samples was observed when considering seawater and gill microbiomes (5.3%), with a value that doubled those observed when comparing ASVs shared between seawater and the other fish microbiome, and even exceeding the percentage of ASVs shared between seawater and sediment microbiomes (4.1%). Considering only fish microbiomes, the gut microbiome shared less than 13% of ASVs with each fish tissue, whereas skin and fillet shared 18.8% of their ASVs (Table 1).

**Table 1:** Summary of ASVs (excluding singletons) shared among different types of samples analyzed in this study, i.e., including both fish- and environment-related microbiomes. Reported are (i) the number of shared ASVs, (ii) the total ASV number, and (iii) the percentage of shared ASVs between pairs of sample types

Type of sample		# of shared ASVs	Total ASVs	% of shared ASVs
Fish gut	Fish fillet	354	2886	12.4
	Fish gill	316	2633	12.0
	Fish skin	410	3459	11.9
	Sediment	39	5243	0.7
	Seawater	74	3386	2.2
Fish fillet	Fish gill	185	1194	15.5
	Fish skin	224	1189	18.8
	Sediment	18	3218	0.6
	Seawater	36	1378	2.6
Fish gill	Fish skin	218	1400	15.6
	Sediment	28	3413	0.8
	Seawater	82	1537	5.3
Fish skin	Sediment	27	3448	0.8
	Seawater	40	1613	2.5
Seawater	Sediment	138	3338	4.1

## Discussion

The microbiome affects many properties of host phenotypes and metabolism [34]. Hence, an in-depth knowledge of fish microbiome is prompted aimed at understanding its role in the host organism [16]. This applies with particular reference to commercially relevant farmed fish species, given the possible implications of the microbiome to their production management, the safety, and quality of fish food produced as well as fish production’s environmental sustainability [9]. Here, for the first time to the best of our knowledge, we report on the different farmed Mediterranean gilthead seabream tissues’ microbiomes and their surrounding environment to test whether different tissues select for different microbial communities and to clarify the extent of the surrounding environment in contributing to the composition of fish-associated microbial communities. Furthermore, we explored the potential interplay among tissues’ microbiomes to shed light on the connections occurring between the different microbiomes and to hypothesize future directions in fish microbiome exploration.

Overall, our study showed clear distinctions between the fish and the surrounding environment microbiomes as well as among the different fish tissues. At the same time, we also observed the presence of connections among fish tissues and the environment. This finding supports the concept that such complex relationships should be studied more in detail and taken into account when designing and applying microbiome-based approaches to improve and manage farmed fish productions. In agreement with previous studies, our results indicate that fish have distinct associated microbiomes compared with the external environment [18, 35], confirming the notion that fish hosts select for specific assemblages, and that host species rather than the surrounding environment is the primary driver shaping microbial communities in fish [36]. Overall, the percentage of shared ASVs between fish microbiomes and the environment was higher with seawater than with sediments, suggesting that connections between fish and the environment mainly occur with the pelagic compartment, as expected for a pelagic species such as *S. aurata*. However, more experimental studies are needed to clarify this point.

Our results showed a higher diversity in environmental samples than in fish microbiomes, in contrast to previous observations [35]; however, it is noteworthy mentioning that different HTS approaches were used (454 vs. Illumina) and that such a discrepancy could have been caused by the different methodological approaches applied. As expected, planktonic communities were composed of typical marine classes such as the Alphaproteobacteria and Cyanobacteria; similarly, benthic assemblages were composed of common surface sediment-associated taxa, such as Gamma-, Delta-, and Epsilonproteobacteria [37]. Additionally, Proteobacteria, Firmicutes, and Actinobacteria, that are generally recognized as the dominant phyla in the fish microbiome with particular reference to the intestinal content and gut microbiome [16], were also similarly dominant in our study. Interestingly, we found lower Bacteroidetes in fish than in environmental samples, in contrast to several studies indicating this phylum as one of the most abundant in fish [16]; however, it is noteworthy mentioning that Bacteroidetes commonly dominate herbivores fish gut microbiomes [38], whereas *S. aurata* exerts a carnivore feeding style, which may explain the such discrepancy. At the ASV level, different sets of dominant ASVs characterized environmental and fish samples, suggesting the presence of discrete and potentially consistent signatures of microbial communities unique to the investigated tissues [39], as well as to the environmental samples. We speculate that such specific signatures may exert a role in the maintenance of the host health, an aspect that surely deserves further clarifications.

A further level of differentiation was observed when considering the environmental and fish microbiomes separately. Overall, as expected, we find higher richness values in sediment than in seawater, with several taxa, including *Vibrio* and *Sulfurovum*, as well as Rhodobacteraceae and *Synechococcus*, being differentially displayed in the two matrices and confirming established previous notions on the composition of benthic and pelagic bacterial communities [40]. Within microbiome fish tissues, higher richness values were found in the gut than in the other fish tissues, as generally reported for sea bream [41, 42]. Based on the evidence that increased microbial diversity is associated with improved physiology and homeostasis in humans [43]; we speculate that our finding supports a key role of gut microbes in fish health. Although gill microbiomes have been generally reported to display a lower richness compared to those of skin and water [17, 18], diversity in seabream skin and gills has been described as not significantly different [3], supporting the results of our study. Moreover, despite a partial overlapping of the overall community composition depicted by the nMDS analysis, the gut microbiome significantly differed from that of gill, skin, and fillet, suggesting that, although in communication with each other, each tissue favors the establishment of a specific community, likely in response to host-specific organizing factors.

Taxonomic characterization of seabream gut showed, in our dataset, the dominance of *Photobacterium*, *Pseudomonas*, and *Staphylococcus* genera, which have been already reported as important taxa in fish microbiomes. *Photobacterium* species are found widespread in marine habitat [44] as pathogens or decomposers of dead fish, as well as commensals in the gut of many marine organisms [45], although some *Photobacterium* species are also found on the skin and gills of marine fish [46, 47]. Notably, *Photobacterium* exerts contrasting behaviors in association with fish, with a member of this genus being associated with healthy individuals [18, 46] and others, such as *Photobacterium damselae* subsp. *piscicida*, representing the aetiological agent of pasteurellosis [48], suggesting an opportunistic lifestyle towards the fish host. The *Pseudomonas* genus was found in the core gut microbiome of 5 sympatrically farmed marine fish species [49] as well as in the core mid-gut microbiota of Mediterranean seabass and seabream [15] whereas *Staphylococcus* epidermidis represented one of the core OTUs in *S. aurata* in the Aegean Sea [15]. *Pseudomonas* members have been often reported in carnivorous teleosts and reported the most abundant shared bacterial species in the gut of *S. aurata* and *D. labrax* [50], and have been reported to contribute to teleosts’ digestion through the secretion of several digestive enzymes [50 and references therein]. Interestingly, *Pseudomonas*, together with *Acinetobacter*, *Psychrobacter*, and *Shewanella*, was also abundant in skin, although different ASVs were associated with the different host tissues.

Among the most represented taxa in skin samples, we found ASVs assigned to the genera *Pseudomonas*, *Acinetobacter*, *Psychrobacter*, and *Shewanella*. Some of these taxa, such as *Acinetobacter*, have been often associated with fish external microbiomes [19, 36]. Interestingly, despite being reported as fish spoilage agents [51], *Pseudomonas* and *Psychrobacter* strains have repeatedly shown their potential as probiotics in aquaculture [52, 53]. Similarly, a strain of *Shewanella* (e.g., SpPdp11), isolated from the skin of healthy specimens of *S. aurata*, is being widely tested as a fish health modulator; the capacity of this strain on wounded skin healing [54], as well as its regulatory role in skin inflammatory and epithelial barrier function [55] has been demonstrated in several fish species. Recently, a tight connection between fish skin-associated microbes and intestinal barrier functioning was suggested in a study investigating the use of this probiotic strain of *Shewanella* in skin-injured gilthead seabream [54].

Gill samples were enriched in ASVs belonging to the genera *Psychrobacter*, *Cobetia*, and *Pseudoalteromonas*. In a recent study by Rosado et al. [3], such taxa were not reported among those most abundantly found in Mediterranean Sea bream gills collected in Portugal. However, considering that our results indicate the gill microbiome as the one displaying the highest similarity with bacterioplankton communities compared to the other tissues, we hypothesize that such a discrepancy may be explained by the differences in the environmental microbial community composition at different sites. Although little is known about the presence of these taxa in gilthead seabream, all these genera were defined among those significantly abundant OTUs in mucosal surfaces (i.e., skin and gills) of healthy individuals in Yellowtail Kingfish [18]. Gills are vital for fish health, as fishes breathe and excrete waste through them, and they are also potential sites of pathogen invasion and colonization by other microbes [17] as well as a barrier preventing pathogen invasion. However, we know little about the gill microbiome and the factors shaping their diversity. Taken together, these results suggest that a set of potential beneficial microorganisms are enriched in the skin and gill microbiomes, with possible implications of the general health of seabream individuals that deserve further clarifications. Lastly, only a little information on fillet-associated microbiome diversity is reported in the literature for seabream [56, 57]. Interestingly, most of the fillet abundant taxa were shared with the other fish microbiomes (e.g., *Photobacterium*, *Staphylococcus*, *Pseudomonas*), and included some taxa previously described as fillet-associated in a study on other finfish species [58]. However, it must be pointed out that, in such studies, the word “fillet” refers to a (minimally) processed food product whose microbiome might have been impacted by contamination occurring during processing and storage. This substantially differs from our fillet samples, which were basically represented by a portion of fish muscle isolated from the entire fish avoiding, to the greatest possible extent, contamination from working surfaces or fish skin. Based on available knowledge, the muscles of healthy fishes are usually considered sterile, despite the debate on this aspect is still open [22]. Indeed, contamination of fish muscles is possible when immunological resistance is compromised [59], which may likely occur in aquaculture settings under stressful conditions. Although a potential, unavoidable cross-contamination between skin and fillet may have occurred during sampling, our results showing the sharing of bacterial ASVs between the fillet and other tissues, such as the gut or gills (which are unlikely to be caused by cross-contamination), highlight the need for more investigations to fully decipher the presence and role of bacteria in fish muscles.

Considering the level of shared ASVs as a proxy of the connections between different microbiomes, we found that, overall, connections were relatively low among the host’s microbiomes (i.e., skin, gut, gill, and fillet) and, in particular, between the gut and the others fish-related microbiomes. Despite further studies are needed to ascertain whether this is an established pattern in seabream, a recent study on fish skin and gut microbiomes reports that skin-associated communities were highly dependent upon environmental factors, conversely to gut-associated assemblages, suggesting that the stability of abiotic conditions within each tissue (e.g., intestinal pH) may have a role in promoting specialization of the microbiomes [39] and likely supports the lower level of ASVs sharing of the gut with the other fish microbiomes. Nevertheless, given the tight link between the fish microbiome and health [60, 61] and the knowledge acquired in the exploration of other animal microbiomes (e.g., human), we hypothesize that the connections between the fish gut and other fish tissues occur not only in terms of exchanges in microbial taxa but also through the production of metabolites by microorganisms associated to the different fish compartments [13]. A preliminary support to this hypothesis has been provided by [18], who showed the link between fish gut health status on the structuring of skin and gill bacterial assemblages. We also report here that skin, gills, and fillet were found to be the most connected microbiomes in seabream, suggesting the possibility of easier communications taking place among these compartments. If, from one side, close connections between skin and gills appear to be expectable, due to their continuous exchange with seawater and the resuspended sediments, more should be clarified about their connection with fillet. Only a small set of ASVs was shared among all the different seabream microbiomes, suggesting that such bacteria might represent the core members of seabream microbiomes and that these members are able to adapt to different conditions. Finally, a lower similarity was observed between the fish microbiomes and the environment. The higher number of ASVs shared between seawater and gills (around 4%) underlines the closer relationship occurring between this mucosal tissue and the surrounding environment and highlights the role of site features in structuring the outer microbiome of fish [39]. Overall, our results suggest that, although at different levels and depending on the tissue, the seabream microbiome is somehow connected with the environment and the surrounding environment acts as a source of microbes for the fish microbiome [8 and references therein].

## Conclusions

The tight link between microbiome composition and fish health is being increasingly explored in teleosts, including the commercially relevant *S. aurata* species. Our understanding of the complex interactions occurring among fish and environmental microbiomes is still limited and, to better clarify all the aspects, dedicated studies including multi-omics able to trace and combine taxonomic and functional contributions are needed. The information here provided on fish microbiome composition according to the different types of tissue (skin, gill, gut, fillet) and its potential relationships with the surrounding environment (seawater, sediment) provides an additional step towards the exploration of the fish microbiome, useful also to implement future microbiome-based applications improving aquaculture practices. We show that the seabream microbiome, despite differentially sharing a number of microbial taxa with the surrounding seawater and sediments, which suggest the occurrence of relationships between the teleost and the surrounding marine microbiome, displays tissue-specific communities with a highly specific microbial signature. Future research is required aimed at better understanding the nature of the interactions occurring among a host’s microbiomes and how this affects fish health.

## Data Availability

Raw sequence reads are available in the NCBI’s SRA—Short Read Archive under accession PRJNA692072.
